# Edentulism and quality of life in the Salvadoran population: a cross-sectional study

**DOI:** 10.1186/s12903-024-04581-3

**Published:** 2024-08-10

**Authors:** Guillermo Alfonso Aguirre Escobar, Francisco José Rivas Cartagena, Wendy Yesenia Escobar de González, Katleen Argentina Aguirre de Rodríguez, Manuel Bravo, Francisco Mesa, Ángel Gil de Miguel, Aida Maricela Gómez de Martínez, Ana Lourdes Pérez Siciliano

**Affiliations:** 1 Epidemiology and Public Health Program, University Rey Juan Carlos, Madrid, 28933 Spain; 2 Research Center, Faculty of Dentistry, University of El Salvador, San Salvador, El Salvador; 3 Professor of Preventive and Community Dentistry School of Dentistry, University of Granada, 18071 Granada, Spain; 4 Department of Periodontics, School of Dentistry, University of Granada, Granada, Spain; 5 Department of Preventive Medicine and Public Health, University Rey Juan Carlos, 28933 Madrid, Spain

**Keywords:** Quality of life, Tooth loss, Epidemiology, Dentistry, Public health

## Abstract

**Introduction:**

Edentulism is the partial or total loss of teeth, it is irreversible and disabling due to its sequelae in the masticatory, phonetic and aesthetic function that affect the quality of life.

**Objective:**

To establish the impact of edentulism and sociodemographic factors on the quality of life of the Salvadoran population.

**Materials and methods:**

Secondary cross-sectional analysis of data in 3322 users of the Public Health System of El Salvador, aged 15 to > 60 years. The variables under study were sociodemographic, edentulismo and quality of life. Edentulism was determined by clinical examination using the Oral Impact on Daily Performance scale. The statistical analysis was performed using χ^2^, OR, multiple regression analysis and set the significance threshold at *p* < 0.05.

**Results:**

Partial edentulism in the upper jaw was present in 68.24% people, partial edentulism in the lower jaw was present in 72.42% people and complete edentulism was observed in 2.02% people. There were significant sex differences and a relationship between sex and quality of life (*p* < 0.004); the self-perception of severe/very severe impacts was greater in women. People without education or with primary or secondary education only were the most affected (*p* < 0.05). Tooth loss increases with age, affecting quality of life in a severe/very severe manner. Complete edentulism had greater impacts on quality of life in terms of eating (25.64%), speaking (21.15%), and socializing/enjoying contact with people (10.90%). A severe/very severe impact on quality of life of teeth lost was reported mainly by those over 60 years of age, with an average of 11 missing posterior teeth, 6 missing anterior teeth and 13 missing teeth per patient. Those missing up to 6 anterior teeth were times more likely to perceive severe/very severe impacts on quality of life than those without any missing teeth (OR:5.788). Edentulism affected the quality of life of those examined, especially the loss of upper anterior teeth.

**Supplementary Information:**

The online version contains supplementary material available at 10.1186/s12903-024-04581-3.

## Background

Edentulism is the partial or total loss of permanent teeth, generally as a consequence of dental caries and periodontal disease [1, 2]. It is an irreversible condition and is currently considered an epidemiological measure of oral health, monitored in many countries and age groups [3–6].

Although other countries have reported significant reductions in the prevalence of tooth loss, this condition still represents a major health problem in the Salvadoran population. According to the most recent National Oral Health Survey in El Salvador [7], adults between 35 and 44 years of age are missing an average of 4 permanent teeth, while Salvadorans over 65 years of age reported a dramatic increase of 16 missing teeth on average.

Because of the extent of its sequelae, the loss of permanent teeth continues to be considered a public health problem that deserves attention. It has been related to a wide range of clinical alterations, such as dental migrations and alterations in the temporomandibular joint, as well as functional disorders that compromise swallowing, phonation, mastication, and aesthetics, among others [3, 8]. In addition, functional and aesthetic sequelae may impact the psychosocial status of the patient, with a number of adverse effects on quality of life [1, 9].

According to the World Health Organization (WHO), quality of life can be understood as “the individual’s perception of his or her life situation, in the sociocultural and value context in which he or she lives in relation to his or her goals, expectations, norms, concerns, which are related to physical health, psychological state, degree of independence, social relationships and religious beliefs“ [10]. Although quality of life is a subjective measure, it is receiving increasing attention in the assessment of patient health, planning of treatment and evaluation of treatment outcomes.

To measure the impact of oral conditions on quality of life, different instruments have been developed, including the Daily Impact Index on Oral Health (OIDP), which was developed and validated by Adulyanon and Sheiham and presented at the Chapel Hill Conference in 1996 [11]. Which has demonstrated adequate psychometrics to be applied in cross-sectional studies, in addition to being validated in several countries and languages [6, 12]. There are no studies with a large national sample that have analyzed the association between oral health and quality of life in El Salvador. Therefore, the purpose of this study was to establish the impact of edentulism and sociodemographic factors on the quality of life of the Salvadoran population through the OIDP index.

## Methods

### Study design and setting

This study complied with the Strengthening the Reporting of Observational Studies in Epidemiology (STROBE) guidelines [13]. The present cross-sectional and analitic study involved a secondary analysis of data derived from a study on the impact on quality of life of the loss of permanent teeth. Patients of both sexes who attended 22 community family health units were selected through convenience sampling. A total of 3322 patients (aged 15 to > 60 years) from the Public Health System of El Salvador participated in the study; patients voluntarily agreed to participate in the study after meeting the inclusion criterion of having lost at least one permanent tooth.

The National Ethics Committee approved the execution of this study. The participation of patients in the research was voluntary, and informed consent or assent was obtained from the parents or legal guardians of included individuals.

### Measurements

The data were recorded by 22 previously trained and calibrated general dentists in 4 practical theoretical workshops and 1 pilot study in order to unify criteria, test instruments, establish average times, achieve adequate consistency in diagnostics and data recording. According to the criteria established in the IOPD for the unification of criteria. The statistical analysis to determine inter-examiner agreement for use of the OIDP was Cohen’s Kappa (0.89). The results were obtained using Epidat 3.1 software.

Data were collected by the examiner using a questionnaire consisting of 3 parts. The first part collected sociodemographic information, such as age, sex, and education level, while the second part contained the OIDP scale, which measures physical, psychological, and social aspects. The physical dimension includes eating, brushing teeth, talking and physical activities; the psychological dimension includes sleeping, smiling and emotional stability; and the social dimension includes work activities and contact with people.

Subjects were questioned about their perceived quality of life in relation to their oral condition. To obtain the effect of each dimension, the score obtained for frequency was multiplied by the severity score, yielding a score on each dimension ranging from 0 to 25 points. Each element was classified according to the score obtained as having “no effect” (0 points), “very slight effect” (1 to 5 points), “slight effect” (6 to 10 points), “moderate effect” (11 to 15 points), “severe effect” (16 to 20 points) or “very severe effect” (21 to 25 points). The total score on the OIDP scale was obtained through the sum of the scores of the eight dimensions; consequently, the score could vary from 0 to 200 points. The overall OIDP index score was then used to classify the impact of patients’ oral health on perceived quality of life as “none” (0 points), “very slight” (1 to 40 points), “slight” (41 to 80 points), “moderate” (81 to 120 points), “severe” (121 to 160 points), and “very severe” (161 to 200 points).

After the interview, the participants were clinically examined according to WHO guidelines [14], and the data were recorded in the third part of the form. Taking into account the respective biosafety measures, the patients were asked to sit in the dental chair to undergo an oral examination. The clinical evaluation was carried out with the aid of a properly sterilized diagnostic set, the loss of permanent teeth was determined, and the prevalence of edentulism was recorded in the observation guide, according to the missing component of the decayed, missing, and filled permanent teeth (DMFT index) (See supplementary file 1).

### Statistical analysis

Data analysis was performed in SPSS version 26.0 (SPSS^®^ Inc., IBM Corp., Armonk, NY, USA) for Windows. Descriptive statistics of variables included the calculation of percentages, means and standard deviations for the sample characteristics. Multiple logistic regression analysis was performed with the OIDP score and the sociodemographic and clinical variables as independent variables. The confidence level was set at 95%. The statistical tests used are described at the bottom of each table.

For the analysis of OIDP scores, the 6 categories of impact on quality of life were recategorized into 3 categories: “no effect/very mild effect”; “mild/moderate effect” and “severe/very severe effect”.

## Results

A total of 3322 subjects participated in the study: equal percentage of men and women. , el 89.15% of whom had some formal education. The mean age was 43 ± 31.36 years. From 34 years of age onward, a slight to moderate impact of oral condition on quality of life was evident.

Regarding clinical variables, edentulism in the upper jaw was reported in 68.24% participants, and edentulism in the lower jaw was reported in 72.42% participants; 2.02% of participants had complete edentulism.

Of the participants, 62.76% reported “no/very slight effect” of edentulism on their quality of life, 29.17% reported a slight/moderate effect, and 8.07% perceived a severe/very severe effect (see Table 1).

**Table 1:** Impact on quality of life according to sociodemographic variables and edentulism status (*N* = 3322)

Variable		Quality of life						Total		*P* value
		No/very slight effect		Slight/moderate effect		Severe/very severe				
		*N*	%	*N*	%	*n*	%	*N*	%	
Sex	Male	1086	65.38	459	27.63	116	6.98	1661	50.0	0.004
	Female	999	60.14	510	30.70	152	9.15	1661	50.0	
Age group	15 to 24 years old	408	74.18	111	20.18	31	5.64	550	16.54	0.000
	25 to 33 years old	385	69.49	137	24.73	32	5.78	554	16.66	
	34 to 42 years old	358	64.39	162	29.14	36	6.47	556	16.72	
	43 to 51 years old	328	59.21	184	33.21	42	7.58	554	16.66	
	52 to 59 years old	313	56.4	186	33.51	56	10.09	555	16.69	
	Over 60 years old	293	52.98	189	34.18	71	12.84	553	16.63	
Education level	No education	202	56.42	127	35.47	29	8.1	358	10.77	0.000
	Primary education	482	55.92	291	33.76	89	10.32	862	25.92	
	Secondary education	624	66.38	256	27.23	60	6.38	940	28.27	
	Bachelor’s degree	573	65.64	227	26	73	8.36	873	26.26	
	Associate degree	49	62.03	20	25.32	10	12.66	79	2.38	
	Graduate school	155	73.81	48	22.86	7	3.33	210	6.32	
Maxillary edentulism	Yes	1338	58.97	734	32.35	197	8.68	2269	68.24	0.000
Mandibular edentulism	Yes	1564	64.95	648	26.91	196	8.14	2408	72.42	0.000
Complete edentulism	Yes	49	73.13	10	14.93	8	11.94	67	2.02	0.027

The confidence level was set at 95%.

Those patients who perceived a severe/very severe impact on their quality of life had posterior tooth loss, although the greatest impact was observed for anterior tooth loss in people aged 15 to 33 years and 34 to 42 years. People between 43 and 59 years of age had combined posterior and anterior tooth loss, and in patients over 60 years of age, there was an increase in tooth loss, regardless of the sector (see Table 2).

**Table 2:** Impact on quality of life according to the number of teeth missing

Age group	Edentulism		Quality of life						
			No/very slight effect		Slight/moderate effect		Severe/very severe effect		*p* value
			*N*	%	*n*	%	*n*	%	
15 to 24 years old(*n* = 550)	Type of edentulism	Partial	408	74.18	111	20.18	31	5.64	ND
		Complete	0	0	0	0	0	0	
	NMPT	1 to 8 teeth	405	74.18	110	20.15	31	5.68	0.872
		9 to 16 teeth	3	75	1	25	0	0	
	NMAT	1 to 6 teeth	408	74.18	111	20.18	31	5.64	ND
		7 to 12 teeth	0	0	0	0	0	0	
25 to 33 years old(*n* = 554)	Type of edentulism	Partial	385	69.49	137	24.73	32	5.78	ND
		Complete	0	0	0	0	0	0	
	NMPT	1 to 8 teeth	383	69.89	135	24.64	30	5.47	0.010
		9 to 16 teeth	2	33.33	2	33.33	2	33.33	
	NMAT	1 to 6 teeth	383	69.51	137	24.86	31	5.63	0.095
		7 to 12 teeth	2	66.67	0	0	1	33.33	
34 to 42 years old(*n* = 556)	Type of edentulism	Partial	357	64.32	162	29.19	36	6.49	0.758
		Complete	1	100	0	0	0	0	
	NMPT	1 to 8 teeth	350	66.04	149	28.11	31	5.85	0.000
		9 to 16 teeth	8	30.77	13	50	5	19.23	
	NMAT	1 to 6 teeth	354	64.36	160	29.09	36	6.55	0.805
		7 to 12 teeth	4	66.67	2	33.33	0	0	
43 to 51 years old(*n* = 554)	Type of edentulism	Partial	324	59.02	184	33.52	41	7.47	0.215
		Complete	4	80	0	0	1	20	
	NMPT	1 to 8 teeth	304	61.29	155	31.25	37	7.46	0.110
		9 to 16 teeth	24	41.38	29	50	5	8.62	
	NMAT	1 to 6 teeth	319	59.51	177	33.02	40	7.46	0.686
		7 to 12 teeth	9	50	7	38.89	2	11.11	
52 to 59 years old(*n* = 555)	Type of edentulism	Partial	307	56.23	184	33.7	55	10.07	0.529
		Complete	6	66.67	2	22.22	1	11.11	
	NMPT	1 to 8 teeth	262	59.01	144	32.43	38	8.56	0.130
		9 to 16 teeth	51	45.95	42	37.84	18	16.22	
	NMAT	1 to 6 teeth	294	57.31	172	33.53	47	9.16	0.033
		7 to 12 teeth	19	45.24	14	33.33	9	21.43	
Over 60 years old(*n* = 553)	Type of edentulism	Partial	255	50.9	181	36.13	65	12.97	0.006
		Complete	38	73.08	8	15.38	6	11.54	
	NMPT	1 to 8 teeth	164	57.54	99	34.74	22	7.72	0.010
		9 to 16 teeth	129	48.13	90	33.58	49	18.28	
	NMAT	1 to 6 teeth	229	54.01	155	36.56	40	9.43	0.000
		7 to 12 teeth	64	49.61	34	26.36	31	24.03	

**NMPT**: Number of missing posterior teeth

**NMAT**: Number of missing anterior teeth

The confidence level was set at 95%.

With regard to specific dimensions of the OIDP index, men reported the greatest impact on eating, smiling and socializing in a normal way, while women reported the greatest impact on talking, sleeping, maintaining an emotional state and enjoying contact with people (see Table 3).

**Table 3:** Quality of life according to dimensions of the OIDP scale

Variable	OIDP index								
	Eating and enjoying food n (%)	Speaking and pronouncing clearly n (%)	Cleaning or brushing teeth n (%)	Sleeping and relaxing n (%)	Smiling, laughing and showing teeth without embarrassment n (%)	Maintaining usual emotional state without being irritable n (%)	Carrying out major work or social role n (%)	Enjoying contact with people n (%)	Total n (%)
Sex									
Male	1094 (25.42)	490 (11.38)	743 (17.26)	198 (4.6)	731 (16.98)	257 (5.97)	361 (8.39)	430 (9.99)	4304 (46.59)
Female	1211 (24.54)	561 (11.36)	852 (17.26)	245 (4.96)	831 (16.84)	347 (7.03)	393 (7.97)	495 (10.03)	4935 (53.41)
Age group									
15–24 years old	307 (25.78)	103 (8.65)	221 (18.56)	55 (4.62)	208 (17.46)	83 (6.97)	96 (8.06)	118 (9.91)	1191 (12.89)
25–33 years old	344 (25.94)	123 (9.28)	252 (19)	60 (4.52)	225 (16.97)	84 (6.33)	111 (8.37)	127 (9.58)	1326 (14.35)
34–42 years old	392 (26.08)	145 (9.65)	290 (19.3)	63 (4.19)	265 (17.63)	87 (5.79)	114 (7.58)	147 (9.78)	1503 (16.27)
43–51 years old	398 (24.33)	183 (11.21)	286 (17.51)	73 (4.47)	289 (17.7)	118 (7.23)	129 (7.9)	157 (9.61)	1633 (17.68)
52–59 years old	432 (24.59)	218 (12.41)	282 (16.05)	95 (5.41)	281 (15.99)	113 (6.43)	149 (8.48)	187 (10.64)	1757 (19.02)
> 60 years old	432 (23.62)	279 (15.25)	264 (14.43)	97 (5.3)	294 (16.07)	119 (6.51)	155 (8.47)	189 (10.33)	1829 (19.79)
Education level									
No education	279 (24.8)	156 (13.87)	174 (15.47)	55 (4.89)	194 (17.24)	74 (6.58)	90 (8)	103 (9.16)	1125 (12.17)
Primary education	655 (24.68)	328 (12.36)	431 (16.24)	126 (4.75)	462 (17.41)	169 (6.37)	209 (7.87)	274 (10.32)	2654 (28.73)
Secondary education	614 (25.92)	281 (11.87)	439 (18.53)	98 (4.14)	407 (17.18)	125 (5.28)	172 (7.26)	233 (9.84)	2369 (25.64)
Bachelor’s degree	577 (24.23)	230 (9.66)	413 (17.35)	122 (5.12)	387 (16.25)	185 (7.77)	217 (9.11)	250 (10.5)	2381 (25.77)
Associate degree	57 (23.36)	19 (7.79)	49 (20.09)	17 (6.97)	36 (14.75)	19 (7.79)	22 (9.02)	25 (10.25)	244 (2.64)
Graduate school	123 (26.39)	37 (7.94)	89 (19.1)	25 (5.37)	76 (16.31)	32 (6.87)	44 (9.44)	40 (8.59)	466 (5.04)
Edentulism									
Partial	2265 (24.94)	1018 (11.21)	1586 (17.47)	432 (4.76)	1545 (17.01)	592 (6.52)	737 (8.11)	908 (9.99)	9083 (98.31)
Complete	40 (25.64)	33 (21.15)	9 (5.77)	11 (7.05)	17 (10.9)	12 (7.69)	17 (10.9)	17 (10.9)	156 (1.69)
Maxillary loss									
Yes	1625 (23.83)	809 (11.87)	1141 (16.74)	333 (4.88)	1185 (17.38)	445 (6.53)	564 (8.27)	716 (10.5)	6818 (73.8)
Mandibular loss									
Yes	1700 (25.22)	748 (11.1)	1145 (16.98)	346 (5.13)	1101 (16.33)	482 (7.15)	553 (8.2)	667 (9.89)	6742 (72.97)

Regarding the quality of life according to the average number of teeth lost, people over 60 years of age were the most affected, i.e., severe/very severe effect, with an average of 13 missing teeth. Individuals aged 25 years and older reported reduced quality of life with losses of 3 or more posterior teeth (see Table 4).

**Table 4:** Impact on quality of life according to the average number of teeth lost

Variable Age		Impact on quality of life			Average number of missing teeth
		No/very slight effect	Slight/moderate effect	Severe/very severe effect	
15 to 24 years old	General loss	2 (1.90–2.09)	2 (1.62–2.37)	2 (1.64–2.35)	2 (1.91–2.08)
	NMPT	1 (0.90–1.09)	2 (1.62–2.37)	1 (0.64–1.35)	
	NMAT	0 (0.00-0.09)	0 (0.00-0.18)	1 (0.64–1.35)	
25 to 33 years old	General loss	2 (1.80–2.19)	3 (2.66–3.33)	4 (2.61–5.38)	3 (2.83–3.16)
	NMPT	2 (1.80–2.19)	2 (1.66–2.33)	3 (1.96–4.03)	
	NMAT	0 (0.00-0.09)	0 (0.00-0.16)	1 (0.30–1.69)	
34 to 42 years old	General loss	3 (2.68–3.31)	4 (3.38–4.61)	6 (4.69–7.30)	4 (3.75–4.24)
	NMPT	3 (2.79–3.20)	4 (3.53–4.46)	5 (4.02–5.98)	
	NMAT	1 (0.89–1.10)	1 (0.69–1.30)	1 (0.34–1.65)	
43 to 51 years old	General loss	4 (3.56–4.43)	6 (5.27–6.72)	7 (5.48–8.51)	5 (4.58–5.41)
	NMPT	3 (2.67–3.32)	5 (4.56–5.43)	5 (4.09–5.90)	
	NMAT	1 (0.78–1.21)	1 (0.71–1.28)	2 (1.09–2.90)	
52 to 59 years old	General loss	7 (6.33–7.66)	8 (7.13–8.86)	11 (9.16–12.83)	8 (7.50–8.49)
	NMPT	5 (4.55–5.44)	6 (5.42–6.57)	7 (5.95–8.04)	
	NMAT	2 (1.66–2.33)	2 (1.56–2.43)	4 (3.21–4.78)	
Over 60 years old	General loss	12 (10.96–13.03)	12 (10.85–13.14)	16 (14.13–17.86)	13 (12.24–13.75)
	NMPT	8 (7.42–8.57)	9 (8.42–9.57)	11 (10.06–11.93)	
	NMAT	4 (3.54–4.45)	3 (2.42–3.57)	6 (5.06–6.93)	

**(Significance level for all variables:** *p* **< 0.05)**

**NMPT: Number of posterior missing teeth**

**NMAT: Number of missing anterior teeth**

The multivariate logistic regression used to identify factors associated with a severe/very severe impact on quality of life is shown in Table 5. Three significant factors were identified: partial edentulism (OR 0.238, 95% CI: 0.1-0.564 and *p* = 0.0010), maxillary edentulism (OR 1.44, 95% CI: 1.078–1.924 and *p* = 0.0140) and the loss of one to six anterior teeth (OR 5.788, 95% CI: 3.678–9.106 and *p* = 0.0000).

**Table 5:** Multivariate analysis of severe/very severe impact on quality of life in the study population

Variable	OR	Confidence interval	*P* value
Maxillary edentulism	1.44	1.078–1.924	0.0140
Loss of 1 to 6 anterior teeth	5.788	3.678–9.106	0.0000
With partial edentulism	0.238	0.1-0.564	0.0010
$$\:{\chi\:}^{2}=105.725$$			

The confidence level was set at 95%.

## Discussion

The present investigation included 3322 users of the public health system, with a mean age of 43 ± 31.36 years, of both genders in equal proportions and almost all users had some schooling. Women reported higher prevalence of a severe/very severe impact on quality of life of edentulism; this difference was significant (*p* < 0.004). Similar to the study by Botello-Harbaumet et al. [15]. on US patients with an average age of 40 years, women were more likely to have a negative self-perception of their quality of life than men (OR = 1.46; 95% CI = 1.06–1.99). Similarly, Caglayan et al. [16]. found that women tended to have a significantly higher negative self-perception of their oral quality of life than men (women 0.86 and men 0.64). De la Fuente-Hernández et al. [17]. found a greater impact on female participants among older Mexican adults. According to the results, females show a greater affectation in quality of life, however, we could not determine the cause, as it was not a study variable. Caglayan et al. [16]. , in Turkey, in respect that the QoL of females appeared to be more susceptible to disruption by oral disorders. De la Fuente Hernandez et al. [17]. , do not comment on the reason for the differences and cite Jimenez et al. [18]. , who comment that women are more concerned about their health status, which is seen in the higher demand for medical care. However, this does not necessarily reflect the fact that men are healthier, a situation that may be due to cultural and gender influences, as it is more difficult for them to accept their limitations and ailments.

In addition, the presence of a severe/very severe impact on quality of life was observed at 15 years of age (5.64%), with an upward trend until the age of 60 years and older (12.84%). A statistically significant difference was established (*p* = 0.000). These results are similar to those of Botello-Harbaum et al. [15]. , who also found a statistically significant association (*p* < 0.001). Likewise, education level had a significant influence (*p* < 0.000). This is similar to the results of Caglayan et al. [16]. in a Turkish population, who reported significant negative perceptions of patients who did not receive formal education or had attended only primary school (*p* < 0.001). These results are probably influenced by the lack of resources to replace missing teeth, given that prosthetic rehabilitation is not part of the programs of most public health services due to the costs involved.

With respect to the type of missing teeth, mandibular, maxillary or complete edentulism, a significant influence on quality of life was found (*p* < 0.000). Similar to the studies of Ogunrinde et al. [3]. in Nigeria, 47.3% of missing teeth were from the upper jaw, with a statistically significant relationship between the location of missing teeth and the severity of the impact on the quality of life of the participants. Batista et al. [5]. reported a consistent impact on quality of life in Brazilian individuals with the number and position of missing teeth, with a prevalence of 48.1%. Sánchez-García et al. [19]. also found a relationship between quality of life and edentulism in Mexico. In Ecuador, Borda et al. [20]. found increased risk according to the number of missing teeth from an OR of 1.35 (95% CI: 0.75–2.43; *p* = 0.32) in older adults with less than 4 missing teeth to an OR of 1.88 (1.06–3.32; *p* = 0.029) in those with more than half of teeth missing. In addition, the number, location, and distribution of missing teeth also affected people’s self-perception. The consistency of results across countries confirms that edentulism affects quality of life; therefore, due to the high costs involved in prosthetic rehabilitation programs, preventive programs that avoid or reduce such losses are needed.

The present study showed that the older the patient is, the greater the increase in the impact on quality of life. There is also evidence that in individuals between 15 and 33 years of age, missing teeth in the posterior had the greatest impact, although the effect on quality of life was greater when anterior teeth were missing, as is the case in patients between 34 and 42 years of age. While a combined loss of posterior and anterior teeth was observed in patients from 43 to 59 years of age, patients over 60 years of age exhibited greater loss of teeth, with a higher proportion of individuals with complete edentulism and corresponding severe/very severe impact on quality of life. In Nigeria, Ogunrinde et al. [3]. found that the majority (47.3%) of missing teeth were from the upper jaw; almost half of the respondents had missing posterior teeth (49.7%), while 37.5% were missing anterior teeth and 12.7% were missing anterior and posterior teeth. Borda et al. [20]. examined edentulism-related quality of life in Ecuador and reported that 77.13% of older adults self-reported fair/poor health depending on the number of missing teeth. In the fourth national survey in China, Gao et al. [21]. reported that in countries such as Canada, the United States, Finland, Sweden, the United Kingdom, Japan, Thailand, Australia and China, the predominant rates of complete edentulism have been maintained at 6–57% in adults over 65 years of age [22]. Regardless of the country affected, we conclude that the loss of anterior teeth has a moderate to severe/very severe impact on the quality of life of its population; therefore, it is important to promote oral health from an early age to prevent tooth loss in adults and thus maintain or improve general health and corresponding quality of life.

Regarding the impact on specific dimensions of the OIDP scale, men reported the greatest impacts on eating, smiling and socializing in a normal way, while women reported the greatest impacts on talking, sleeping, maintaining emotional state and enjoying contact with people. From the age of 34 years onward, eating and brushing teeth are the most affected dimensions, while after 60 years of age, there is greater difficulty in eating and pronouncing words correctly. University students with tooth loss reported the greatest impacts on eating and socializing in a normal way, while participants with no formal education reported the greatest impacts on speaking and pronouncing words; individuals with primary education only reported the great impacts on smiling and showing their teeth. Complete edentulism had a greater impact on people’s quality of life in the domains of eating, speaking, socializing and enjoying contact with people, while partial edentulismo, in one of the jaws, mainly affected eating, smiling, brushing teeth and speaking. In Greece, Papaioannou et al. [23]. found that oral health impacted quality of life, observing significant correlations with functional limitation (*p* < 0.01), handicap (*p* < 0.05) and social disability (*p* < 0.01). In Ecuador, Borda et al. [20]. reported that edentulism mainly impacted eating and enjoying food, cleaning or brushing teeth, and smiling or showing teeth. In Brazil, Saintrain and De Souza [24] reported that 81.9% of the study population experienced difficulties after tooth loss, affecting the physical, psychological and social dimensions. In Mexico, Sánchez-García et al. [19]. found that the main problems reported by persons over 60 years of age were eating (14.4%), speaking (8.7%), inability to avoid irritation (5.4%), brushing teeth (5%) and inability to enjoy contact with people (4.4%). The above studies consistently indicate that the most affected dimension in all populations was functional limitations, i.e., eating and enjoying food, since the loss of natural teeth restricts the type of food consumed and reduces the supply of nutrients necessary for the body, which leads to general health problems.

In our study, the impact on quality of life varied according to the number of missing teeth, mainly affecting people over 60 years of age, since they were missing up to 11 posterior teeth and 6 anterior teeth, with an overall average loss of 13 teeth, which severely/very severely affected their quality of life. Quality of life was affected from the age of 25 years onwards, with the loss of 3 posterior teeth. From 34 to 51 years of age, the average number of missing teeth was 5 posterior teeth and 2 anterior teeth; this number increased from 52 to 59 years of age, with an average of 7 missing posterior teeth and 4 missing anterior teeth; this difference was significant (*p* < 0.05). In Brazil, Batista et al. [5]. reported that 48.1% of the population studied reported quite/very frequent impairment in one or more daily activities due to edentulism. Significant prevalence rate ratios (PRRs) for severity were obtained for those missing up to 12 teeth, including one or more anterior teeth (PRR = 1.63, 95% CI 1.06–2.51); those missing 13–31 teeth (PRR = 2.33, 95% CI 1.49–3.63); and those with complete edentulism (PRR = 2.66, 95% CI 1.55–4.57) compared to adults without any missing teeth.

The multivariate analysis indicated that the Salvadoran study population with maxillary edentulism was 1.44 times more likely to have a severe/very severe impact on quality of life compared to patients without maxillary edentulism. Patients missing up to 6 anterior teeth were 5.78 times more likely to report a severe/very severe impact on quality of life than those without any missing anterior teeth. Partial edentulism reduced the likelihood of reporting a severe/very severe impact on quality of life to 23.8% (OR = 0.238) compared to patients with complete edentulism.

One of the limitations of this study is that the data cannot be extrapolated to the entire population but only to users of the Salvadoran public health system. The results obtained will serve to develop specific oral health care programmes to reduce edentulism and thus counteract the impact on the quality of life of the most affected population groups. In addition, dental schools should emphasise the repercussions of edentulism on quality of life in their different programmes, especially in terms of the impact on masticatory function. And the country’s health authorities should prioritise the development of public policies and specific oral health strategies aimed at the most vulnerable population. Future research on the subject should include the causes of the differences in perception of the impact on quality of life between men and women and differences between age groups.

## Conclusions

Eating or enjoying food and showing the teeth were most affected aspects in terms of the quality of life of the edentulous population studied, mainly in those missing upper anterior teeth, leading to the perception of a severe/very severe impact on quality of life. The older the patient was, the greater the loss of teeth and the greater the impact on quality of life. The prevalence of partial edentulism was higher than that of complete edentulism; however, people with complete edentulism were the most affected, highlighting the need for specific public health programs aimed at this population.

### Electronic supplementary material

Below is the link to the electronic supplementary material.

Supplementary Material 1

## Data Availability

The datasets generated and analyzed during the current study are not publicly available due to ethical limitations concerning anonymity but are available from the corresponding author on reasonable request.

## Abbreviations

WHO: World Health Organization
OIDP: Oral Impact On Daily Performance
STROBE: Strengthening the Reporting of Observational Studies in Epidemiology
DMFT: Decayed, Missing, and filled permanent teeth index
SPSS: Statistical Package for the Social Sciences
PRR: Prevalence Rate Ratios
OR: Odds Ratio
CI: Confidence interval
