# Supplementary material for: Edentulism and quality of life in the Salvadoran population: a cross-sectional study
Source: BMC Oral Health. 2024 Aug 10;24:928. doi: 10.1186/s12903-024-04581-3 (PMC11317007; doi:10.1186/s12903-024-04581-3)
Supplement: Supplementary file 1 — Supplementary Material 1 [file 12903_2024_4581_MOESM1_ESM.pdf]

UNIVERSITY OF EL SALVADOR
FACULTY OF DENTISTRY

OBSERVATION GUIDE

1. SOCIODEMOGRAPHIC VARIABLES:

Purpose: To record the sociodemographic variables of the users of the Community Family Health Units (CFHU) that are part of the study.

Indications:

1. Orient the questions directly to the interviewee in a punctual and understandable manner.
2. Indicate in the blank box on the right the corresponding answer with a blue pencil. The questions are closed-ended, so you should select only one answer per question.
3. Indicate with an asterisk (*) in items 8 and 9 the corresponding answer.

1. Date of Examination: [ ] Day [ ] Month [ ] Year

2. CFHU: [ ]

3. Examiner Code: [ ]

4. Instrument code: [ ]

5. Date of Birth: [ ] Day [ ] Month [ ] Year

6. Sex [ ] 1 Male / 2 Female

7. Edad: [ ]

8. Age group		9. Level of Schooling	
a) 15 to 24 years old		a) No schooling	
b) 25 to 33 years old		b) Primary	
c) 34 to 42 years old		c) Secondary	
d) 43 to 51 years old		d) High School	
e) 52 to 59 years old		e) Technician	
f) Over 60 years old		f) University	

2. IMPACT ON QUALITY OF LIFE VARIABLE/ OIDP INDEX

Objective: To collect information on the ability of the subjects under study to perform their usual activities in the last six months through the OIDP index.

Indications:

1. Orient the questions in Table 1 directly to the interviewee in a timely and understandable manner.
2. Ask each of the questions, recording in box 1 whether or not the interviewee has been affected.
3. In box 2 "Frequency", record the frequency with which the respondent is affected, according to what the respondent indicates. You should consider the possible answers indicated in the same box.
4. In box 3 "Severity", record the severity with which you are affected according to what the respondent indicates. You should consider the possible answers indicated in the same box.
5. The questions are closed-ended, so you should select only one answer per question.
6. At the end, to obtain the effect for each dimension, this is obtained by multiplying the score obtained for the frequency by the score for the severity; and therefore, the score for each dimension is 0 to 25 points. Each element is classified according to the score obtained as follows: "no effect" (0 points), "very slight" (1 to 5 points), "slight" (6 to 10 points), "moderate" (11 to 15 points), "severe" (16 to 20 points) and "very severe" (21 to 25 points).
7. The overall effect of the OIDP is obtained through the summation of the score of the eight dimensions; consequently, the score can vary from 0 to 200 points. The overall OIDP score is then classified as: "no effect" (0 points), "very slight" (1 to 40 points), "slight" (41 to 80 points),

"moderate" (81 to 120 points), "severe" (121 to 160 points), "very severe" (161 to 200 points). Indicate in Table 2 the overall effect of the patient's OIDP.

OIDP INDEX (*Oral Impacts on Daily Performances)*

Table 1

1		2 FREQUENCY		3 GRAVITY		Result of multiplying frequency by gravity
**In the past six months have you had any problems with your mouth, teeth or dentures that cause problems with the following?**		**During the past six months, how often did you have difficulty with the following?**		**Using a scale from 0 to 5, where 0 indicates the absence of a problem in your daily life ("not at all") and 5 indicates the presence of a problem in your life activities ("very serious"), how did it affect your daily life for the following?**		
1. Eating and enjoying food.		1. Eating and enjoying food.		1. Eating and enjoying food.		
2. Speak and pronounce correctly.		2. Speak and pronounce correctly.		2. Speak and pronounce correctly.		
3. Cleaning or brushing teeth.		3. Cleaning or brushing teeth.		3. Cleaning or brushing teeth.		
4. Sleep and relaxation.		4. Sleep and relaxation.		4. Sleep and relaxation.		
5. Smile/laugh and show teeth without embarrassment.		5. Smile/laugh and show teeth without embarrassment.		5. Smile/laugh and show teeth without embarrassment.		
6. Maintain the emotional state, normal and without anger.		6. Maintain the emotional state, normal and without anger.		6. Maintain the emotional state, normal and without anger.		
7. Do all work or socialize in a normal manner.		7. Do all work or socialize in a normal manner.		7. Do all work or socialize in a normal manner.		
8. Enjoying contact with people.		8. Enjoying contact with people.		8. Enjoying contact with people.		
Answers: 0 (YES), 1 (NO)		Responses: (0) Never affected in the last 6 months, (1) Less than once a month or up to five days total, (2) Once or twice a month or up to 15 days total, (3) Once or twice a week or up to 30 days total, (4) Three or four times a week or up to 3 months total, (5) Almost every day or more than three months.		Responses: (0) None, (1) Very little, (2) Little, (3) Moderate, (4) Serious, (5) Very serious.		Dimension score: (0) no effect, (1 to 5) very slight, (6 to 10) slight, (11 to 15) moderate, (16 to 20) severe, (21 to 25) very severe.

Table 2

**OIDP OVERALL EFFECT/ Sum of the 8 dimensions**		
0 points	No effect	
1 to 40 points	Very light	
41 to 80 points	Light	
81 to 120 points	Moderate	
121 a 160 puntos	Severe	
161 a 200 puntos	Very serious	

3. DIAGNOSIS OF REFERRED DENTAL MORTALITY

Objective: To identify the missing dental organs, main clinical and functional alterations.

Indications:

1. Place the patient in the dental chair.
2. Based on the P component, indicate on the odontogram with blue pen the missing dental organs in the oral cavity.
3. Using Table 1, identify the dental organs that after the evaluation are indicated for extraction considering the codes. Identify with red pen the dental organs indicated for extraction.
4. Identify and mark with a blue date the dental organs that present mesialization due to tooth loss.
5. Identify and mark with a blue date the dental organs that present distalization due to tooth loss.
6. Circle in blue the dental organs that are extruded due to loss of their antagonist.

7. Indicate in table 1 and 2 the total number of teeth indicated for extraction, mesialized, distalized and extruded.

1-7	1-6	1-5	1-4	1-3	1-2	1-1	2-1	2-2	2-3	2-4	2-5	2-6	2-7

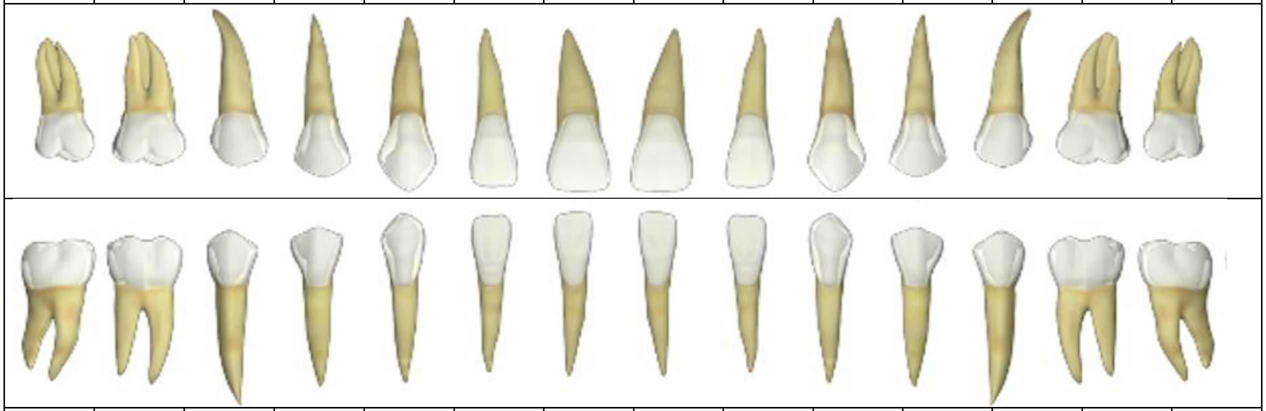

4-7	4-6	4-5	4-4	4-3	4-2	4-1	3-1	3-2	3-3	3-4	3-5	3-6	3-7

Table 1

Teeth indicated for extraction		TEETH	TOTAL
**Code**	**Diagnostic Criteria**		
1	Caries		
2	Periodontal Disease		
3	Trauma		
4	Other causes (orthodontics and prostheses)		

Table 2

	Dientes	Total
**Component P**		
**Indicated for extraction**		
**Mesialized teeth**		
**Distalized teeth**		
**Extruded Teeth**		

4. FUNCTIONAL ALTERATIONS

Indications:

1. According to the clinical evaluation, record in Table 3 the presence or absence of articular noises.

Table 3

Joint noises	Presence	
	Absence	
	Bilateral	
	Unilateral	
	Not applicable	

5. PROSTHETIC CONSIDERATIONS

According to the clinical evaluation, classify according to Kennedy the edentulism presented by the patient, indicate classification in Table 4.

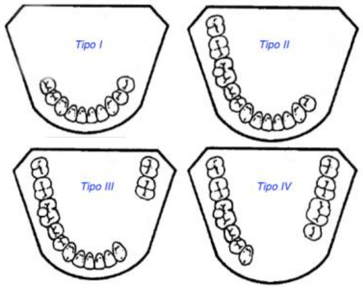

Table 4

Kennedy Classification			
**UPPER MAXILLA**		**LOWER MAXILLA**	
Class I		Class I	
Class II		Class II	
Class III		Class III	
Class IV		Class IV	
Edentulous		Edentulous	
Not applicable		Not applicable	

According to the clinical evaluation, indicate in Table 5 the state or type of prosthesis the patient presents.

Table 5

UPPER MAXILLA		LOWER MAXILLA	
Toothed		Toothed	
Removable Partial Prosthesis		Removable Partial Prosthesis	
Complete Prosthesis		Complete Prosthesis	
Fixed Prosthesis		Fixed Prosthesis	
Not Applicable/No prosthesis		Not Applicable/No prosthesis	

Remarks:____

____
